# Anomaly Detection Method for Rocket Engines Based on Convex Optimized Information Fusion

**DOI:** 10.3390/s24020415

**Published:** 2024-01-10

**Authors:** Hao Sun, Yuehua Cheng, Bin Jiang, Feng Lu, Na Wang

**Affiliations:** 1 College of Automation Engineering, Nanjing University of Aeronautics and Astronautics, Nanjing 211106, China; rogerduke@nuaa.edu.cn (H.S.);; 2 College of Energy and Power Engineering, Nanjing University of Aeronautics and Astronautics, Nanjing 210016, China

**Keywords:** rocket engine, data fusion, convex problem, fault diagnosis

## Abstract

The power system, as a core component of a launch vehicle, has a crucial impact on the reliability and safety of a rocket launch. Due to the limited measurement information inside the engine, it is often challenging to realize fast and accurate anomaly detection. For this reason, this paper introduces the rocket flight state data to expand the information source for anomaly detection. However, engine measurement and rocket flight state information have different data distribution characteristics. To find the optimal data fusion scheme for anomaly detection, a data set information fusion algorithm based on convex optimization is proposed, which solves the optimal fusion parameter using the convex quadratic programming problem and then adopts the adaptive CUSUM algorithm to realize the fast and accurate anomaly detection of engine faults. Numerical simulation tests show that the algorithm proposed in this paper has a higher detection accuracy and lower detection time than the traditional algorithm.

## 1. Introduction

As a complex aerodynamic–thermal system, the rocket engine works in a high temperature, high pressure, and strong oxidizing environment, and the structural performance of its components may be degraded and malfunction, which may lead to the fault of the engine system. Therefore, the engine is a sensitive and frequently failing part of the launch vehicle, which is a severe threat to the reliability and safety of rocket launch missions [1,2]. Monitoring the operation status of the rocket power system and discovering anomalies in time can prevent the impact of non-fatal failures on the rocket transportation mission and improve the safety and reliability of the launch vehicle.

Early studies for liquid rocket engine fault diagnosis were mainly based on signal processing methods [3,4]. For example, the anomaly and failure detection system (SAFD) proposed in the 1980s [5], the flight accelerometer safety shutdown system (FASCOS) [6], is mainly based on the red line threshold alarm method. With the development of science and technology, artificial-intelligence-based fault diagnosis methods are widely used in various nonlinear and complex systems since they only need to make full use of prior knowledge and information about the diagnosis object. Wheeler et al. built a sensor model of the main engine of the space shuttle based on a radial basis neural network for fault diagnosis [7]. YU proposed a method to optimize the BP (back propagation) using adaptive genetic algorithms and neural networks for real-time fault detection algorithm for liquid rocket engines [8]. Tsutsumi for maintenance of reusable liquid propellant rocket engines, fault detection was carried out using bivariate time-series analysis, where the state of individual sensors was extracted using principal component analysis, based on which the sensor faults of liquid rockets were estimated [9].

The internal sensors of the rocket power system are the fastest to characterize the faults. Still, the limited number of sensors inside the system due to the limitation of the internal space and the harsh measurement environment and installation conditions makes it difficult to realize accurate and fast diagnosis by solely relying on the engine data. The output thrust of the engine can directly affect the flight state of the rocket, so the information of the flight state and attitude control system can also reflect the working condition of the engine. The fusion of engine internal measurement information, flight state information, and attitude control information provides an effective engine anomaly detection method [10].

Data fusion schemes are categorized into three types: data-level, feature-level, and decision-level fusion. In recent years, scholars have conducted many studies on fusion-based anomaly detection schemes [11].

The data-level fusion scheme has been repeatedly used by scholars in the design of algorithms for anomaly detection due to its ability to retain the original information. Azamfar et al. addressed the problem of fault detection in gearboxes by fusing data from multiple current sensors through a two-dimensional convolutional neural network architecture, which can be directly used for classification without manually extracting the time-frequency features of the data, and finally verified by the current data of industrial gearboxes, which showed that the proposed method has the best classification performance [12]. The proposed method has the best classification performance in fault detection [12]. Jiang conducted research on the uncertainty information in sensor data fusion and proposed a method combining Z-number and D-S (Dempster-Shafer) evidence theory. The method can model the fuzzy and reliable information of sensor data and fuse them to provide a more robust reliability metric for sensor data, which improves the reliability of fault detection [13]. Liu et al. proposed a method for constructing a comprehensive health index by fusing multiple degradation-based sensor data. The method improves the degradation-based prognostic model through the steps of data selection, processing, and fusion, which ultimately better describes the operational status of the system [14].

Since the feature-level fusion scheme has less computational overhead due to the ability to extract key information, it is gradually being widely used in fault diagnosis. Buchaiah et al. extracted 72 original features from bearing vibration data using signal processing techniques and selected a subset of relevant features from the extracted features using the Random Forest method [15]. The selected features were finally fused by dimensionality reduction to search for the most effective rolling bearing fault diagnosis technique. Jing et al. proposed a data fusion method based on a deep convolutional neural network for feature layer sensors for the problem of damage detection in complex mechanical systems. The method learns features from raw data, adaptively optimizes different combinations of fusion levels, and finally meets the requirements of the fault diagnosis task [16]. Radman et al. proposed a fusion scheme based on D-S evidence theory for the problem of epileptic seizure detection. The scheme extracts features from the EEG (Electroencephalogram) signals, analyzes the features by Pearson correlation coefficient, and finally realizes feature-fusion-based epilepsy detection by an ensemble decision tree classifier [17].

As all kinds of systems become more complex, decision-level data fusion schemes are more often applied to anomaly detection for their advantage of handling multimodal data. Xu et al. proposed a multi-model decision fusion method based on deep convolutional neural networks and improved D-S evidence theory. Through this method, the features of the data can be extracted and input into the network model for decision fusion, and the damage detection of rolling bearings is finally realized [18]. Chao et al. proposed a multi-sensor fusion method for axial piston pump faults, considering the limited sensor data. The method uses a convolutional neural network to accept vibration data from three channels and makes the final diagnosis by fusing the information at the decision-making layer [19]. Grbovic utilizes a sparse principal component analysis algorithm to decompose the process monitoring sensor network into multiple blocks and generates local predictions using SVM (Support Vector Machine). Afterwards, the maximum entropy algorithm is used to fuse the local prediction results, which finally realizes the distributed fault detection of the sensor network [20].

In summary, decision-level fusion applies to multimodal scenarios, feature-level fusion applies to heterogeneous data scenarios, and data-level fusion realizes fusion based on raw data, which can maximally retain data information [21,22]. Therefore, this paper is oriented to the needs of fault detection and selects the data-level fusion algorithm to realize the data fusion of the launch vehicle. In addition, scholars have carried out many studies on various types of data fusion schemes. Still, most studies utilize intelligent algorithm training to obtain the fusion parameters without going through the mathematical derivation process, which may not result in the optimal fusion effect [23]. The data composition is more complex since the rocket power system and control system include sensor measurements and control instructions. Therefore, in this paper, we choose the optimized data-level fusion scheme to realize the anomaly detection of the rocket power system, which will not lose the fast information of the engine, can deal with multimodal communication, and is easy to debug. Since the fused data can already characterize the current system, considering the real-time demand of the rocket power system detection, this paper chooses the faster statistical-based data flow detection algorithm.

## 2. Preliminary

### 2.1. Research Population

The kinetic equations of the launch vehicle are as follows [24,25]:

(1) $$\begin{array}{l} m\frac{dV}{dt}=P\cos\alpha \cos\beta -mg\sin\theta +X_{2c}+X_{2e}\\ mV\frac{d\theta }{dt}=P\left(\begin{array}{l} \sin\alpha \cos\gamma_{v}+\\ \cos\alpha \sin\beta \sin\gamma_{v} \end{array}\right)-mg\cos\theta +Y_{2c}+Y_{2e}+F_{By}\\ -mV\cos\theta \frac{d\sigma }{dt}=P\left(\begin{array}{l} \sin\alpha \sin\gamma_{v}-\\ \cos\alpha \sin\beta \cos\gamma_{v} \end{array}\right)+Z_{2c}+Z_{2e}+F_{Bz} \end{array}$$

where *m* represents the mass of the launch vehicle, V represents the velocity of the rocket, P represents the thrust of the rocket, $X_{2c},Y_{2c},Z_{2c}$ represents the control force of the three channels of the launch vehicle, $X_{2e},Y_{2e},Z_{2e}$ represents the inertia force due to the engine swing, α and β represent the head-on and side-slip angles, θ and σ represent the velocity inclination and velocity deflection, $\gamma_{v}$ represents the velocity roll angle, and $F_{By},F_{Bz}$ represents the disturbance force.

**Figure 1:**
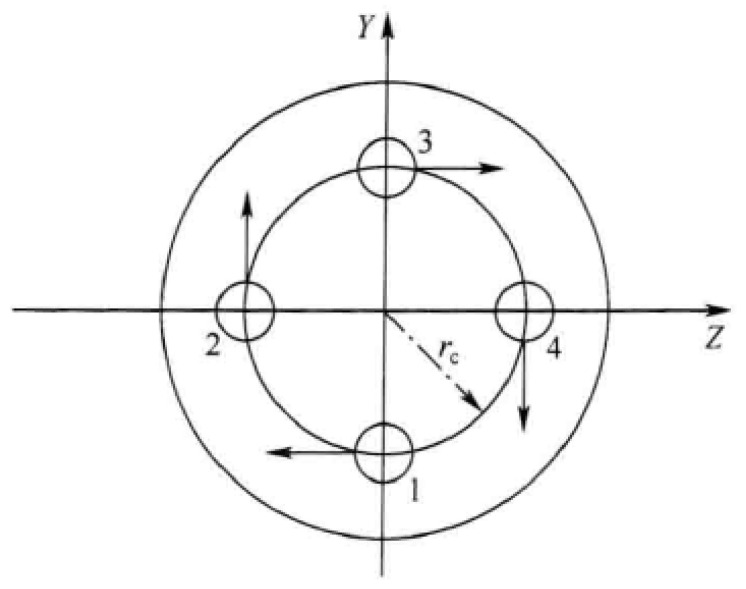
Gimbaled rocket engine configuration.

The engine numbering sequence and the positive direction of the swing angle are shown in Figure 1, and the direction of the engine swing angle is defined as positive clockwise deflection when viewed from the tail end of the rocket [26]. When the swing engine swing angle is $\delta_{1},\delta_{2},\delta_{3},\delta_{4}$, the equivalent swing angle of the three channels can be regarded as

(2) $$\begin{array}{l} \delta_{\varphi }=\left(\delta_{4}-\delta_{2}\right)/2\\ \delta_{\psi }=\left(\delta_{3}-\delta_{1}\right)/2\\ \delta_{\gamma }=\left(\delta_{1}+\delta_{2}+\delta_{3}+\delta_{4}\right)/4 \end{array}$$

The control torque generated by the engine oscillation is

(3) $$\{\begin{array}{l} M_{x1c}=-Pr_{c}\delta_{\gamma }\\ M_{y1c}=-\frac{P}{2}x_{c}\delta_{\psi }\\ M_{z1c}=-\frac{P}{2}x_{c}\delta_{\varphi } \end{array}$$

where $x_{c}$ is the distance from the engine rocking axis to the center of mass of the rocket and $r_{c}$ is the distance from the engine rocking axis to the longitudinal axis of the arrow body.

**Figure 2:**
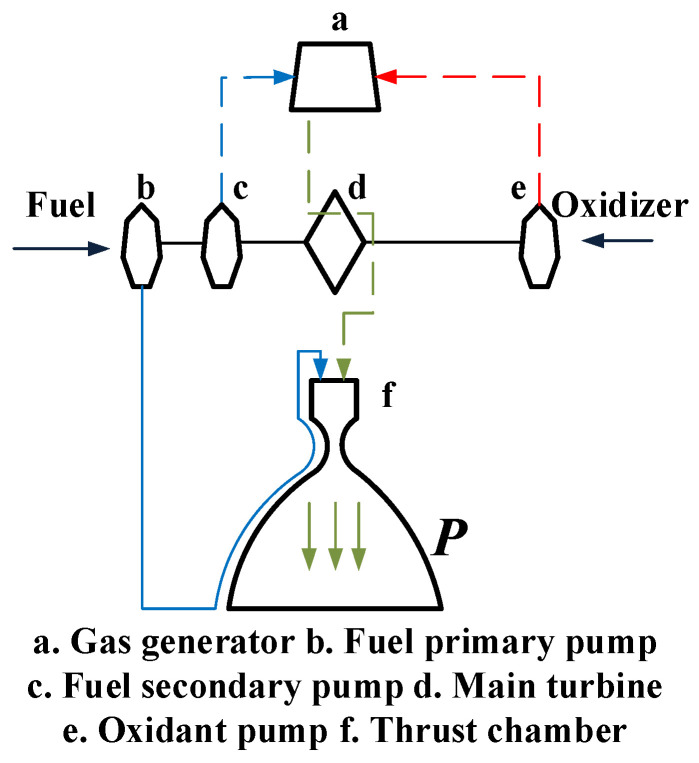
Principle of operation of liquid rocket engine.

The rocket engine model shown in Figure 2 mainly consists of the main turbine pump, a pre-pressurized turbine pump, a gas generator, a thrust chamber, valves, piping, and other components, liquid oxygen through the oxygen pre-pressurized turbine pump, the oxygen main pump, and the liquid oxygen main valve into the gas generator; the generator produces a high-temperature oxygen-rich gas to drive the main turbine after the gas conduit into the combustion chamber after the make-up combustion [10]. Fuel kerosene is pressurized by the fuel pre-pressure pump and the fuel primary pump and divided into four ways, most of which enters the combustion chamber through the cooling channel of the thrust chamber, a part of which flows into the gas generator and the ignition path of the thrust chamber after increasing the pressure by the fuel secondary pump, another part of which drives the fuel pre-pressure pump and then flows into the main path, and the last part of which drives the servo mechanism.

From the above, in the rocket flight process, engine failures such as turbine blade ablation failure or pump cavitation failure will directly affect the thrust; thrust changes will affect the control force, control torque, etc., and ultimately act on the flight attitude control system, affecting the flight attitude of the rocket.

To establish the engine component failure model, this paper adopts the method of multiplying the parameters of the engine component mathematical model by a specific coefficient (the coefficient is called the failure factor) [3]. In the event of engine component failure, the mathematical model of the engine component can be expressed as follows:

(4) $$\{\begin{array}{l} \frac{dX_{e}}{dt}=H\left(X,U,D*F,t\right)\\ Y=G\left(X,U,D*F,t\right) \end{array}$$

where $X_{e}={\left[x_{e1},x_{e2},\cdots ,x_{em}\right]}^{T}$ is the state parameter of the component module, $U={\left[u_{1},u_{2},\cdots ,u_{m}\right]}^{T}$ is the input parameter of the component module, D is the parameter of the mathematical model of the component module, t is the time, $Y={\left[y_{1},y_{2},\cdots ,y_{m}\right]}^{T}$ is the output parameter of the component module, H(·) and G(·) are the functional relations in the component module to construct the connection between the above parameters, and F is the fault factor, which characterizes the severity of the component fault.

### 2.2. Existing Problems

Before using the data-level fusion method to solve the problem of the accurate real-time monitoring of the abnormal state of the rocket power system, this paper carries out an in-depth analysis of the data of the control system and the power system to clarify the difficulty of the problem and target the design of the algorithm.

**Figure 3:**
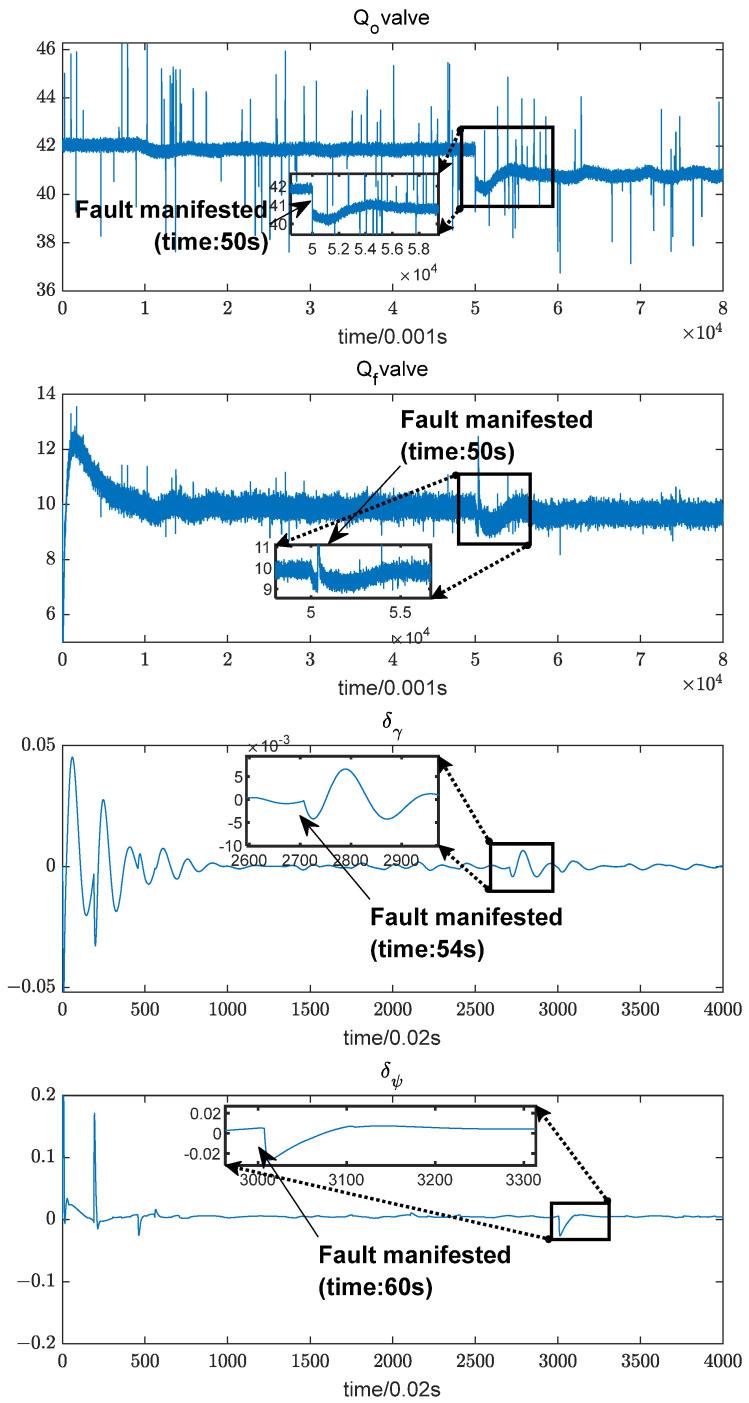
Comparison of rocket multi-system parameters.

A typical fault, the oxidizer pre-pressurized turbine blade ablation fault, is used as an example to compare the parameters of the two systems at 50 s when the fault occurs. As shown in Figure 3, the engine parameters can characterize the occurrence of the fault in time. Still, its internal parameters fluctuate considerably, affecting the characterization of the abnormal state inside the power system. The control system parameters are slower to characterize the fault but are more stable and can indirectly reflect the thrust change. In summary, integrating power system and control system parameters can accurately and quickly characterize the occurrence of faults.

Although fusion schemes for data may fuse multimodal data and improve the performance of anomaly detection, this paper discusses the fusion between data from different systems, which may pose some potential challenges. There are several difficulties in performing the parameter fusion of rocket power systems and control systems for anomaly detection as follows: 

1. The information is highly coupled: the engine’s measurement information and flight state information require different time lengths for fault characterization; on the other hand, due to the dynamics, these information are highly coupled with each other, which is an important challenge for fusion algorithms;

2. Strengthen the fault characteristics: to achieve accurate detection, the fusion algorithm also needs to find the optimal fusion parameters and strengthen the fault characteristics in order to improve the accuracy of the anomaly detection algorithm, which requires that the fusion algorithm can be targeted to the fault characteristics in the most obvious way to fuse the data.

3. High real-time requirements: in some engine failure cases, the power system will quickly fail or even explode, so the anomaly detection algorithm requires high real-time performance.

Given the above problems, this paper proposes an anomaly detection scheme based on convex optimization data fusion from the data characteristics of Rocket stabilization flight parameters. The method can find the optimal fusion scheme to strengthen the fault characteristics and meet the real-time and accuracy requirements of anomaly detection. The remaining parts of this paper are composed as follows: the third part introduces the anomaly detection algorithm design and process of this paper, the fourth part presents the experimental design of this paper and the results of algorithm validation, and the fifth part summarizes and looks forward to the research content of this paper.

## 3. Method

Based on the problem posed in the previous section, and considering that anomaly detection needs to maximize the difference in data characteristics before and after a fault, the problem is first formulated through two fusion objectives as follows:

Objective 1: The fused data should remain stable in the absence of faults, i.e., the data fluctuation in the absence of faults should be minimized, denoted as $\min\;\mathrm{var}(X_{i})$;

Objective 2: The fusion data should show the maximum change after a failure, i.e., the fluctuation of the fusion data after a failure should be the largest, denoted as $\max\;\frac{dL_{i}}{dt}$.

Based on the above two data fusion objectives and considering the real-time computation demand, this paper selects the optimal fusion parameter as the optimization variable of the fusion problem. After solving this variable, the data can be weighted directly at the data level to obtain the fusion data.

Considering that the measurement noise and fault characterization speed of the launch vehicle engine data and the control system parameters differ, the optimal fusion parameter is based on the global optimum property of convex optimization.

Convex optimization problems are widely used in the spacecraft field due to their nature—“local optimum is global optimum” [27]. Benedikter, for the launch vehicle ascent trajectory optimization problem, used a combination of successive convexification techniques to generate a series of convex problems and a three-step successive procedure to improve the computational performance [28]. Deaconu, for the relative trajectory of spacecrafts under the prior successive constraints on impulsive maneuver-based proximity operations, used semi-positive definite programming to obtain an optimal solution [29]. Harris proposed a real-time solution based on lossless convex optimization for the problem of optimizing the maximum steering trajectory of a planetary landing by transforming the problem constraints into convex constraints [30]. In summary, when a problem can be transformed into problem constraints with convexity, the properties of convex optimization can be considered to obtain an optimal solution.

In this paper, we utilize convex quadratic programming to solve the problem of data fusion between the power system and the engine of a launch vehicle, so that the sensor dataset of the rocket before the failure is denoted as $X_{i,j,k}$, and the dataset after the failure is denoted as $L_{i,j,k}$, where i,j,k correspond to the number of experiments, the number of measurement points, and the number of parameters, respectively.

Through the problem design in this section, the power system data are fused with the relatively smoother control system information by using convex optimization solving to strengthen the fault characteristics. Solving the optimal fusion parameters through the global-optimality property of convex optimization maximizes the difference between the data before and after the fault to improve the effect of anomaly detection.

### 3.1. Convex Optimization Problem Construction

From the above, the fusion of power system and control system information in a launch vehicle requires the data-level fusion of data with different distribution characteristics, and different speeds of fault manifestation, and to obtain the optimal fusion effect for fast and accurate anomaly detection, the fusion problem is formulated as a convex quadratic programming problem.

According to the two fusion objectives mentioned in the previous section, the first fusion objective aims to reduce the fluctuation of the data. In this paper, the variance is used to measure the fluctuation of the data. The variance of the data is estimated through the matrix S and a quadratic form is constructed based on this to ensure the convexity of the constructor.

The second fusion objective is to maximize the change in the failure data, which is measured by the first order difference in the data after the failure. In this paper, we use the matrix $E_{i}$ to evaluate the amount of change in the data and utilize the constraints of the parameter $\gamma_{ij}$ so that the fused data show an overall increasing trend after a fault.

After constructing the fusion objective, this paper introduces the hyperparameter $\alpha ,\beta ,c_{ij}$ to optimize the solution strategy, where α and β are used to balance the importance of the two objective terms, and $c_{ij}$ is used to adjust the weight of the post-fault data.

By combining the two fusion objectives, forming an objective function, and solving it, the optimal fusion parameter can be obtained, which is able to make the optimal combination of multiple parameters with different noise, distribution characteristics, and fault characterization speeds to maximize the fault characteristics.

From the above, the objective function of the convex optimization problem proposed in this paper is as follows. Through this objective function, the parameters of the power system with large observation noise and obvious fault characterization can be fused with the parameters of the control system with better observation quality in order to maximize the differentiation of the data before and after the fault.

(5) $$\begin{array}{c} \underset{w}{\min}\alpha \sum_{i=1}^{n}w^{T}X_{i}^{T}S_{i}X_{i}w+\underset{\gamma_{ij}}{\max}\beta \sum_{i=1}^{n}\sum_{j=1}^{o_{i}-1}c_{ij}\gamma_{ij}\\ \;s.t.w^{T}1=1,E_{i}W_{i}\leq 0\\ \;i=1,\ldots ,n,\;j=1,\ldots ,o_{i} \end{array}$$

where α and β are hyperparameters to adjust the solution strategy of the optimization algorithm.

The first term in Equation (5) is the fusion objective 1, which evaluates the fluctuation of the fused data by estimating the variance of the fused data and ensures the convexity of the objective function by constructing a quadratic form; the second term of Equation (5) is the fusion objective 2, which combines the constraint term $E_{i}W_{i}\leq 0$ to maximize the difference in the post-fault data. There are three hyperparameters in Equation (5), where α and β act together to adjust the importance of the two optimization objectives, and $c_{ij}$ acts together with the parameter $\gamma_{ij}$ to adjust for differences in the post-fault data. The hyperparameters α and β are obtained by empirical analysis, and the sum of these two parameters is a constant, so the appropriate parameter ratio can effectively adjust the optimization effect.

In the first term of Equation (5), w is the fusion weight matrix, which determines the weights corresponding to each set of parameters in the fusion. $X_{i}$ represents the set of rocket data without faults used in training, where the rows correspond to the collected data and the columns correspond to a parameter. $o_{i}$ is the number of measurement points of the post-fault data. S matrix is a symmetric matrix of the following form:

(6) $$S_{i}=\frac{\left(I-\frac{J}{n_{i}}\right)}{n_{i}-1}$$

where I is a unit matrix, J is an all-1 matrix, and $n_{i}$ represents the number of data points collected in the absence of faults.

The matrix S is elicited to estimate the variance of the fused data; the principle is shown in the following equation:

(7) $$\begin{array}{l} \left({\left(X_{j}w\right)}^{T}X_{j}w-n_{i}{\left(\left(1^{T}X_{j}w\right)/n_{i}\right)}^{2}\right)/\left(n_{i}-1\right)\\ =w^{T}X_{j}^{T}\left(\left(I-11^{T}/n_{i}\right)/\left(n_{i}-1\right)\right)X_{j}w/\left(n_{i}-1\right)\\ =w^{T}X_{j}^{T}SX_{j}w \end{array}$$

$c_{ij}$, in the second term of Equation (6), serves as a hyperparameter that determines how much the post-fault data affect the overall optimization objective, and $\gamma_{ij}$ is used to maximize the difference between adjacent data after a fault.

In the constraint term, maximizing the second term in the above equation has the following form:

(8) $$E_{i}=\left[\begin{array}{cccc} \left(l_{i,n_{i}+1,1}-l_{i,n_{i}+2,1}\right) & \cdots & \left(l_{i,n_{i}+1,s}-l_{i,n_{i}+2,8}\right) & \\ \vdots & \ddots & \vdots & I^{i}\\ \left(l_{i,n_{i}+o_{i}-1,1}-l_{i,n_{i}+o_{i},1}\right) & \cdots & \left(l_{i,n_{i}+o_{i}-1,8}-l_{i,n_{i}+o_{i},8}\right) & \end{array}\right]$$

where $l_{i,j,k}$ is the number of measurement points j and sensors k corresponding to the ith experiment; $t_{i}$ represents the number of post-fault data used for training; and $W_{i}={\left[w,\gamma_{i,1},\ldots ,\gamma_{i,o_{i}-1}\right]}^{T}\in R^{\left(8+o_{i}-1\right)\times 1}$ is used to determine the value of $\gamma_{ij}$.

In order to prove the convexity of the proposed information fusion problem, Equation (5) can be formulated as follows:

(9) $$\begin{array}{c} \underset{w,p}{\min}\;\;\;\;\alpha w^{T}Qw-\beta p^{T}I\\ \;s.t.w^{T}1=1,\;\;\;\;\;EW\leq 0. \end{array}$$

where $Q=X^{T}SX,X={\left[X_{1},X_{2},\ldots ,X_{n}\right]}^{T}$, $p={\left[p_{1},p_{2},...,p_{n}\right]}^{T}$, and where $p_{i}={\left[\gamma_{i,1},\gamma_{i,2},\ldots ,\gamma_{i,o_{i}}\right]}^{T}$. Here, the E,W matrix has the same composition as the p. Thus, $S=T\cdot T^{\prime }$, where T is the lower triangular matrix. According to the Cholesky decomposition, the matrix Q can therefore be rewritten as $Q=X^{\prime }SX=(T^{\prime }X{)}^{\prime }T^{\prime }X$, and it is easy to obtain that the Q matrix is also a semi-positive definite matrix.

Thus, the second term in Equation (9) consists of a linear function, while its constraint term is polyhedral and can consist of linear inequalities, making the convex optimization problem easier to solve.

### 3.2. Algorithm Design

**Figure 4:**
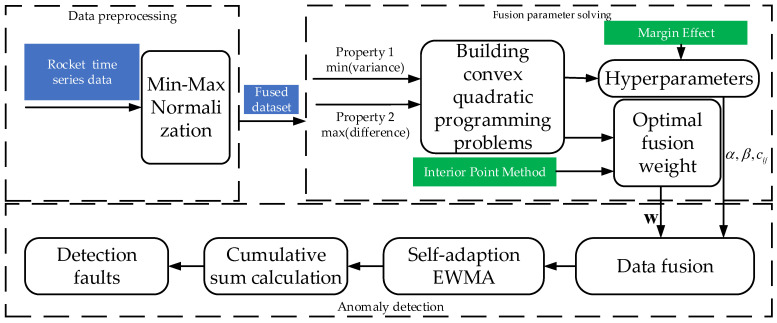
Experimental structure design.

As shown in Figure 4, the detection algorithm flow includes several parts, including data preprocessing, fusion parameter solving, and data stream anomaly detection, and the following parts will be introduced separately next.

#### 3.2.1. Data Preprocessing

Different rocket sensors have different operating frequencies and data characteristics, affecting the fused data quality. Through the data pre-processing link, each sensor data conversion will have different characteristics to achieve the fusion of multiple sensor data. Data pre-processing mainly includes resampling and standardization, enabling data fusion with varying frequencies of sampling and eliminating the scale differences between different data [31].

In this paper, we simplify the problem of fusion of multimodal data and do not consider the problem of data reception and alignment in real situations, but focus on the design of the optimal fusion scheme. The power system data with higher sampling frequencies are resampled so that they can be trained and fused with the control system data.

Since constructing a convex optimization problem requires matrix-positive definitions, which need the final fused data set to have positive covariates, normalization is used to restrict the data to a range of 0–1 to obtain optimal fusion [32].

#### 3.2.2. Fusion Parameter Solving

In order to compute the optimal fusion parameters, the hyperparameters set in Section 3.1 need to be solved first. The post-fault data are stored in $E_{i}$ for training, and, again, the $W_{i}$ matrix and $\gamma_{ij}$ are closely related, and the hyperparameters $c_{ij}$ are solved in training by the constraint term $E_{i}W_{i}$. Combined with the pre-failure data stored in the first term, these are jointly solved to obtain the optimal fusion parameter W. The optimal fusion parameter w is calculated by the constraint term $E_{i}W_{i}$.

In the previous section, the hyperparameters $\alpha ,\beta ,c_{ij}$ of the objective function have not been determined. In order to determine the value of α,β, set α=0,β=1−α initially, and then all parameter choices are traversed with a step size of 0.1 to find the optimal α and β parameters by searching for marginal effects.

The parameter $c_{ij}$ determines how much the post-fault data affect the overall optimization objective. Set $c_{i,j}\geq c_{i,j+1}\geq 0,\forall i,j$ to detect the occurrence of a fault more quickly, in which case $c_{ij}$ has the following form:

(10) $$c_{ij}=c_{i,r_{i}+1}+\left(j-r_{i}-1\right)\frac{2\left(1-t_{i}c_{i,r_{i}+1}\right)}{t_{i}\left(t_{i}-1\right)}$$

The objective function of Equation (5) forms a multi-objective optimization problem through the introduction of three hyperparameters: the first one aims to enhance the smoothness characteristics of the fused data in the absence of faults, and the second one aims to enhance the variability characteristics of the data after faults. The optimal hyperparameters of the multi-objective optimization problem are found by marginal effects through different configurations of the α,β parameters.

After solving the obtained hyperparameters, the optimal fusion parameter can be solved using the interior point method to solve the optimization problem in the previous section. The interior-point method is a numerical optimization method for solving convex optimization problems by searching for a solution within the interior of the feasible solution to avoid the large number of iterative steps that may occur in many exterior-point methods.

The algorithm was first proposed by Karmarkar for solving linear programming problems, and was gradually extended to solve nonlinear convex optimization problems, including second-order cone programming and semidefinite programming.

The basic idea of the interior point method can be formulated as [33]; for a convex optimization problem, the problem can be formulated as finding the minimum value of a convex function f(x) within a certain range of values A. Suppose the following problem is solved:

(11) $$\begin{array}{c} \min f(x)\;\;\;\;x\in A\\ s.t.\begin{array}{c} f_{i}(x)\leq b_{i},\;\;\;\;i=1,2,\cdots ,m\\ h_{i}(x)=0,\;\;\;\;i=1,2,\cdots ,p \end{array} \end{array}$$

The range A is then determined by the constraints in the above equation, i.e., x∈A is equivalent to the constraints.

Next, construct a barrier function g(x) whose function value outside the range A is much larger than the function value inside the range, satisfying

(12) $$g(x)=\{\begin{array}{ll} 0 & x\in A\\ \infty & x\notin A \end{array}$$

The above equation is a function in an ideal state, and a perfect barrier function satisfying this property usually does not exist, so some function is used to make an approximation. A feasible logarithmic type of barrier function is

(13) $$g(x)=\mu \sum_{i}^{m}\ln\left(f_{i}\left(x_{i}\right)\right),\;\;\;\;i=1,2,\cdots ,m$$

where μ is a very small positive parameter, and the range of values of x in g(x) can be in the complex domain.

At this point, the constructor h(x)

(14) h(x)=f(x)+g(x)

When g(x) meet the conditions of the Formula (12), h(x) in the range of *A* outside the function value is much larger than the value of *A*, so this new function h(x) is characterized by the value of the entire domain and can be taken; the minimum value and the minimum value of the value of the range must be in the scope of *A*. At the same time, due to the function of h(x) in the range of *A* and due to the fact that the original value of f(x) is equal, the minimum value of h(x) is the minimum value of f(x). So the optimization problem (Equation (11)) is equivalent to the following problem:

(15) minh(x)    x∈C

where *C* is the complex domain.

This is equivalent to taking the constraints in the initial problem and including them in the functional properties of the newly constructed function h(x). The solution space of the new function is unbounded; thus, the minimum value of the new function h(x) can be found directly using Newton’s method, and the obtained solution is just equal to the minimum solution of f(x) in the original problem within a certain range *A*, i.e., it is the optimal solution of the original optimization problem within the constraints.

After obtaining the hyperparameter $\alpha ,\beta ,c_{ij}$, the optimal fusion parameter w of the data is obtained by solving the optimal solution of the convex quadratic programming problem, at which time the final fused data can be solved using the following equation:

(16) $$T_{f}=\sum_{k}w_{k}l_{i,j,k}$$

#### 3.2.3. Anomaly Detection

The optimization objective of the optimization problem constructed in this paper is to maximize the anomalies of normal and faulty data, and considering the real-time demand of launch vehicle anomaly detection, the detection algorithm should be able to quickly compute to achieve the detection and be sensitive to faults appearing in the fusion data. This paper uses the Exponentially Weighted Moving Average-Cumulative Sum (EWMA-CUSUM) algorithm to realize anomaly detection based on fused data [34]. This algorithm can monitor the data flow in real-time, has slight computational complexity and adaptability, has the property of adjusting the smoothing parameters over time, can always keep monitoring in time-varying situations, is suitable for the characteristics of fused data in engine fault detection, and can realize rapid diagnosis.

When monitoring fusion data, define

(17) $$\begin{array}{c} Z_{t}(\beta )=\left({\hat{\beta }}_{t}-\beta \right)/\sigma \\ Z_{t}(\sigma )=\Phi^{-1}\left\{F\left((n-p){\hat{\sigma }}_{t}^{2}/\sigma^{2};n-1\right)\right\} \end{array}$$

where $\Phi^{-1}\{\cdot \}$ is the inverse standard deviation normal distribution function and F(·;v) is the distribution function of the chi-square distribution ($\chi_{v}^{2}$) with υ degrees of freedom. Such a transformation leads to the design of control charts whose control limits do not depend on the sample size n. Another advantage is that the distribution of $Z_{t}(\sigma )$ is symmetric, so that the control charts will also be quite sensitive to decreases in the variance of the contours. Recall that the vector ${\left(Z_{t}(\beta ),Z_{t}(\sigma )\right)}^{T}$ is a multisource normal random vector with expectation 0 and variance array $\Sigma =\left(\begin{array}{cc} {\left(X^{T}X\right)}^{-1} & 0\\ 0 & 1 \end{array}\right)$.

In calculating the CUSUM control curve, the training sequence mean value u is first obtained, and the smooth parameter is defined as a function of time as follows:

(18) $$g(t)=\{\begin{array}{ll} \begin{array}{ll} g(t)=\lambda , & t=1\\ \max\left(\frac{\lambda }{3-0.99^{t}},\frac{\lambda }{2+0.99^{t}}\right),U_{t}\geq u, & t>1 \end{array}\\ \min\left(\frac{\lambda }{3-0.99^{t}},\frac{\lambda }{2+0.99^{t}}\right),U_{t}<u, & t>1 \end{array}$$

After that, the adaptive EWMA sequences based on GT and ZT are calculated

(19) $$J_{t}=g(t)Z_{t}+(1-g(t))J_{t-1}$$

where $J_{0}=0,\;g(t)\in (0,1]$.

Finally, the adaptive EWMA-CUSUM sequence is computed

(20) $$EC_{t}=\max\left(0,J_{t}^{\prime }\Sigma^{-1}J_{t}-g(t)+{\mathrm{EC}}_{t-1}\right)$$

where ${\mathrm{AMEC}}_{0}=0$, this control chart will signal the runaway alarm in case of $EC_{t}>h$.

It is common to use Average Run Length (ARL) to measure the performance of control charts to monitor linear contour data, with controllable ARL (ARL_0_) and uncontrolled ARL (ARL_1_) defined as follows:

(21) $$\begin{array}{c} {\mathrm{ARL}}_{0}=\frac{1}{\alpha_{c}}\\ {\mathrm{ARL}}_{0}=\frac{1}{1-\beta_{c}}, \end{array}$$

where $\alpha_{c}$ is the probability that the control chart sends a false alarm, and $\beta_{c}$ is the probability that the control chart has a missed detection.

The control limits of the control chart are obtained by bisection search: (1) Fix ARL_0_, and set an upper limit $h_{\max}$ and a lower limit $h_{\min}$, in which the upper limit can be set slightly larger, in order to avoid failing to find the control limits; the lower limit is usually taken as 0. (2) The initial control limit h is set to the upper limit, i.e., $h=h_{\max}$, and the ARL is obtained by statistical simulation:

- If $\mathrm{ARL}\leq {\mathrm{ARL}}_{0}$, raise the lower limit, i.e., $h_{\min}=h$;
- If $\mathrm{ARL}\geq {\mathrm{ARL}}_{0}$, lower the upper limit, i.e., $h_{\max}=h$;
- If ${\mathrm{ARL}}_{0}-1\leq \mathrm{ARL}\leq {\mathrm{ARL}}_{0}+1$, jump out of the loop and find the desired control limit h; otherwise, reset the control limit h, i.e., $h=\frac{h_{\max}+h_{\min}}{2}$.

The control limit T in this paper is determined by the distribution of the training dataset.

## 4. Experiment

### 4.1. Experiment Design

**Figure 5:**
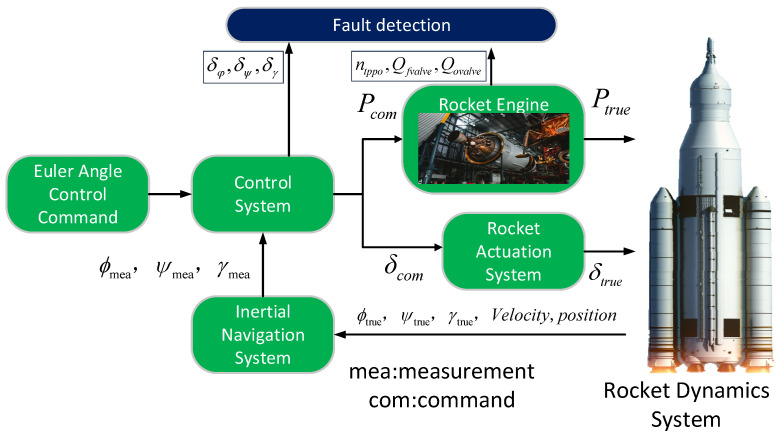
Rocket digital simulation system.

This paper validates the proposed algorithm using the constructed rocket simulation loop data. The rocket simulation loop completely simulates the navigation, guidance, and control systems during the rocket movement, as shown in Figure 5. Combined with the engine system simulation, the time series data of the control system and power system can be obtained during the flight.

The engine simulation takes a liquid oxygen supplementary combustion rocket engine as the research object, including a turbopump, gas generator, combustion chamber, pipeline, valve, and other components. The simulation takes the commanded thrust as the input and the engine thrust, specific impulse, and other performance parameters and internal monitoring parameters of the components as the output so that the engine operating characteristics under the fault state can be simulated.

Considering that the engine data can more quickly characterize the occurrence of faults, three parameters, namely the fuel main valve flow $Q_{fvalve}$, oxygen pre-pressure turbine speed $n_{tppo}$, and liquid oxygen main valve flow $Q_{ovalve}$, are selected to be added into the fusion data set; meanwhile, five parameters, namely the three-axis attitude angle $\delta_{\varphi },\delta_{\psi },\delta_{\gamma }$ and the lateral and normal overloads $n_{y},n_{z}$ are selected to be added into the fusion data set among the parameters of the control system, and the fusion data set is composed as follows:

(22) $$L=[n_{tppo},Q_{fvalve},Q_{ovalve},\delta_{\varphi },\delta_{\psi },\delta_{\gamma },n_{y},n_{z}]$$

The simulation time is set to 80 s and the combustion chamber throat ablation fault is injected inside the engine at 50 s. This fault is more frequent in the rocket engine, which may lead to the damage of the combustion chamber structure, the spalling of the material, and even the failure of the rocket engine. So this fault, as a typical fault of the engine, has a certain degree of representativeness to carry out research on it.

After the fusion data set is selected, the simulation platform is used to construct the training data; in this paper, through Monte Carlo simulation, the random fault factors and attitude control objectives (initial attitude and end attitude) are obtained to form twenty-five groups of data sets. Five groups in the dataset are selected as the training dataset and twenty as the test dataset. The dataset scenarios are configured, as shown in the table below. The parameter ranges for Monte Carlo sampling to simulate the real environment are demonstrated in Equation (23). Different fault factors affect the degree of faults, while different attitude angle control instructions change the trajectory and control scheme of the rocket, which makes the simulation stochastic and allows the verification of the generalization of the algorithm.

(23) $$\begin{array}{c} F\in [0.8,1]\\ \delta_{\varphi \_c}\in [40{}^{\circ},90{}^{\circ}]\\ \delta_{\psi \_c}\in [0{}^{\circ},10{}^{\circ}]\\ \delta_{\gamma \_c}\in [0{}^{\circ},5{}^{\circ}] \end{array}$$

### 4.2. Experiment Results

**Figure 6:**
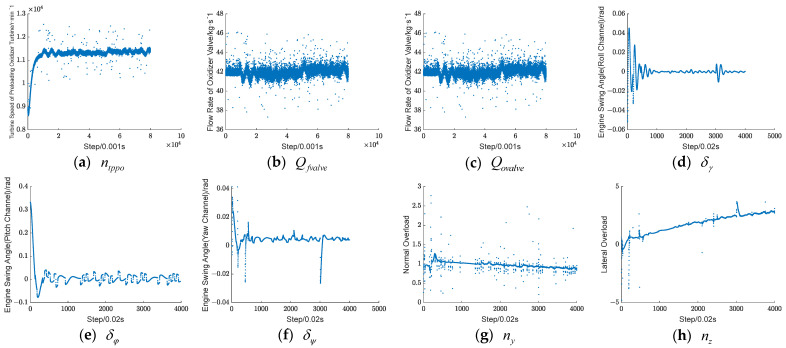
Parameter dataset before fusion.

Figure 6 illustrates the variation of the fused dataset under an oxygen pre-pressurized turbine ablation fault. Since the operating characteristics of the control and engine systems result in different sampling steps for their data, the data from the engine system are resampled and then normalized. As can be seen from the Figure 6, after the fault occurs at 50 s, the parameters all fluctuate to different degrees.

**Figure 7:**
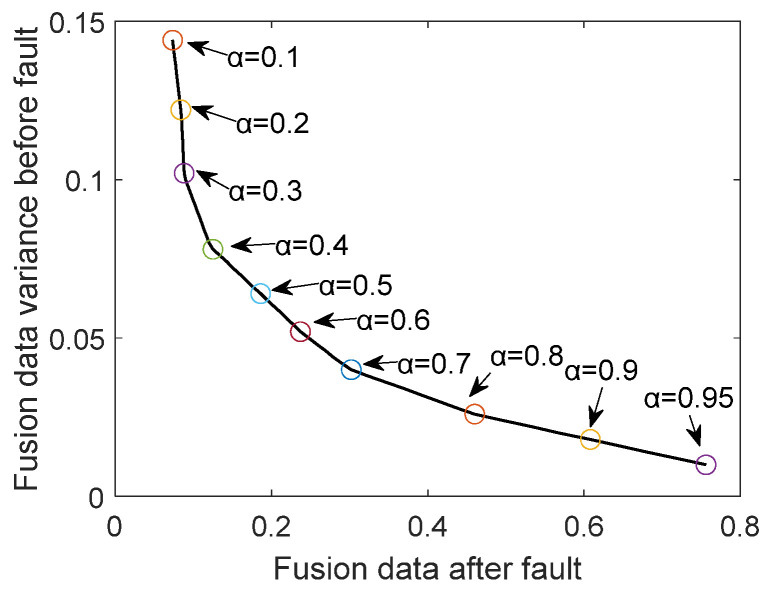
Using marginal effects to determine α.

After data preprocessing, the optimal fusion parameters can be obtained by constructing a convex quadratic programming problem and solving it. The change process of the hyperparameter α mentioned above using the marginal effect to solve is shown in Figure 7. Since α can reflect the importance of the two components in the optimization objective function, this paper chooses α=0.7, because, at this time, the variance decline of the pre-fault data begins to slow down, and the choice of this parameter can achieve the maximum gain of the objective function.

After determining the hyperparameters, the optimal parameters are obtained by solving the optimization problem, and the optimal fusion weight parameters obtained in this paper are shown in Table 1.

E.g., the eight parameters in the table are: Turbine Speed of Oxidizer Pre-pressurized turbopump, Flow Rate of Fuel Valve, Flow Rate of Oxidizer Valve, Engine Swing Angle (Roll Channel), Engine Swing Angle (Pitch Channel), Engine Swing Angle (Yaw Channel), Normal Overload, and Lateral Overload.

**Figure 8:**
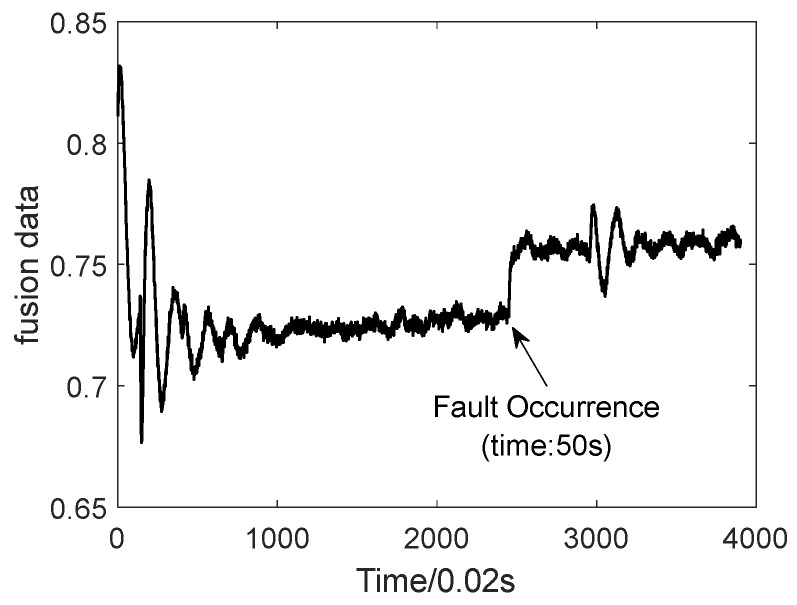
Fused data.

The test dataset is fused based on the optimal fusion parameters and the fused data are shown in Figure 8. Anomaly detection algorithms are designed based on the fused data in the above figure to achieve fast and accurate monitoring. Based on the fused data, the adaptive EWMA-CUSUM algorithm is utilized for the anomaly detection of the fused data, and the upper and lower detection limits in the EWMA-CUSUM algorithm are set as follows:

(24) $$\begin{array}{l} T_{\max}=\mu +2\sigma \\ T_{\min}=\mu -2\sigma \end{array}$$

where μ and σ represent the mean and standard deviation from the first 500-point estimates of the fusion data, respectively.

**Figure 9:**
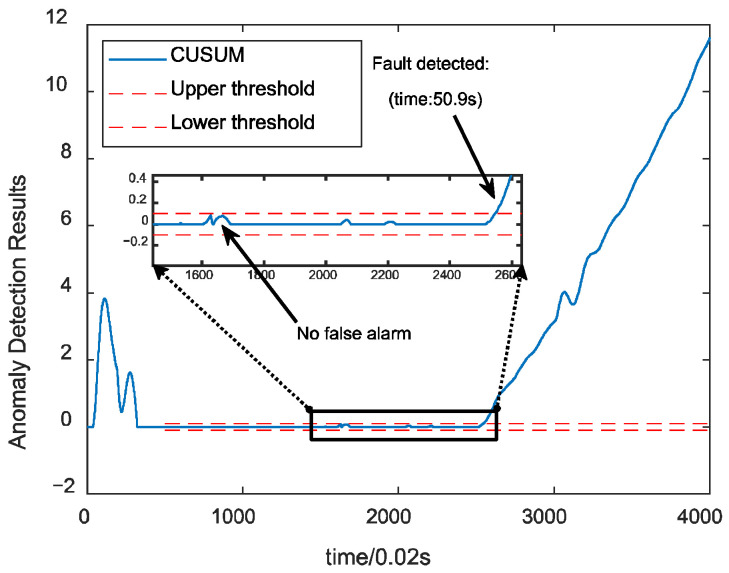
Fused data CUSUM control chart detection results.

**Figure 10:**
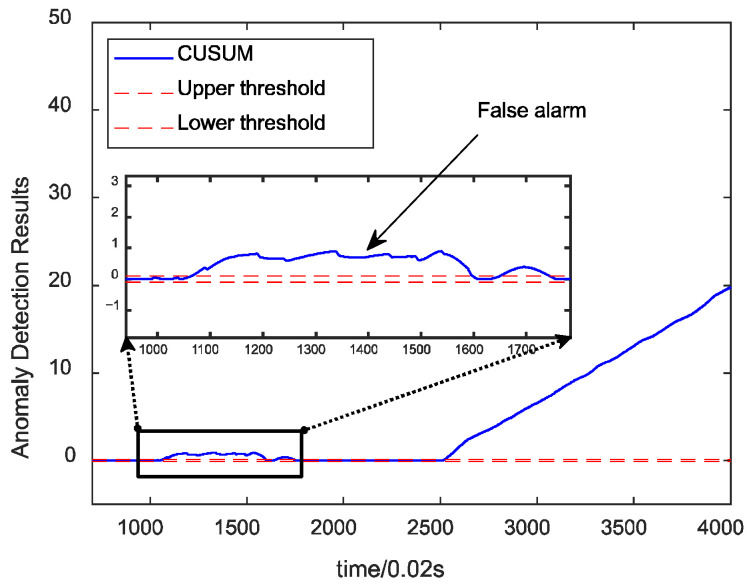
Single-parameter CUSUM detection results.

Figure 9 illustrates the fault detection results for the fused data under the combustion chamber throat ablation fault. The algorithm detects the fault without false alarm, with a time of 0.9 s. Figure 10 demonstrates the results of anomaly detection using a single parameter. From the above figure, the fault can be detected relatively quickly but has a false alarm rate of 32.9%. This indicates that, by fusing the information from the engine and control system, the fluctuation of the data is effectively reduced, and the characteristics of the faults are strengthened, improving the detection results.

**Figure 11:**
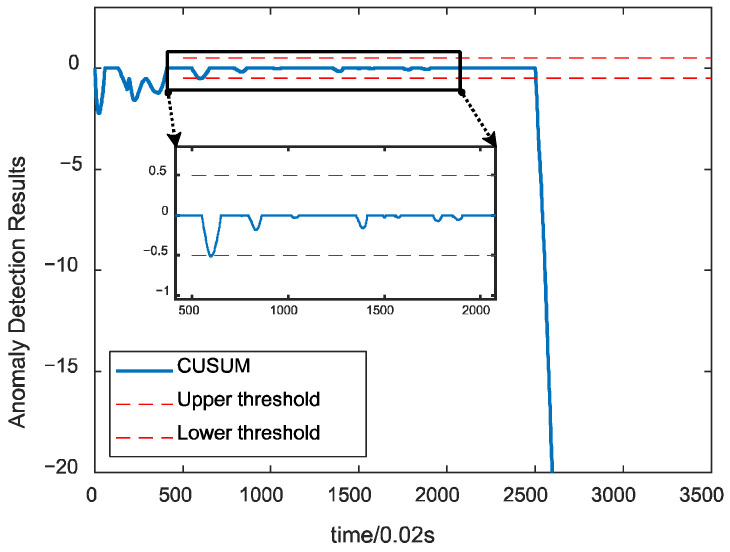
Detection results under main turbine blade erosion faults.

**Figure 12:**
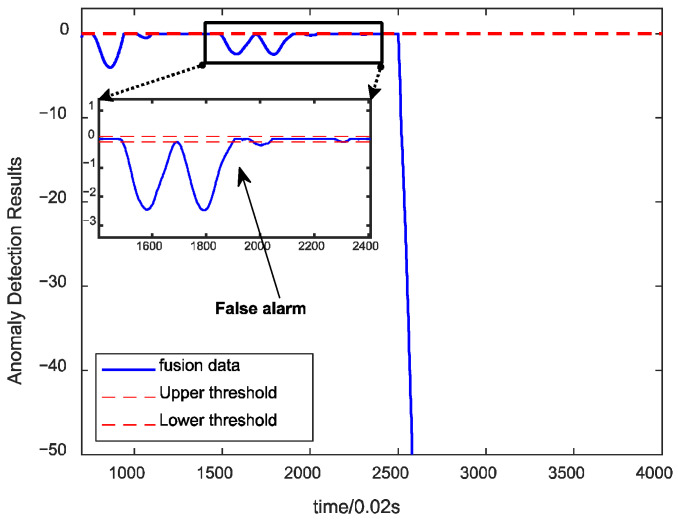
Detection results of single parameter under main turbine blade erosion.

To test the effect of the algorithm under different faults, the proposed algorithm is utilized to detect the fusion data under the main turbine blade ablation fault. The detection results are shown in Figure 11, which shows that the algorithm has no false alarms under this fault, and the detection speed meets the real-time requirements, indicating that the algorithm has sound detection effects on different spots and has good generalizability. Figure 12 shows the detection effect of using a single parameter under the same fault, and there will be missed detection under various faults.

Finally, this paper compares the effectiveness of the proposed algorithm with the commonly used algorithms in fault diagnosis, including classification-based and adaptive thresholding anomaly detection algorithms, under combustion chamber faults. Currently, the mainstream algorithm in the field of liquid rocket fault diagnosis is an anomaly monitoring scheme based on intelligent algorithms. Soon-Young et al. [3] proposed a deep-learning-based fault detection method for the start-up phase of LRE using long-term and short-term memory and CNN (Convolutional Neural Network). Li et al. [35] combined PSO (Particle Swarm Optimization) with LSSVM (Least Squares Support Vector Machine), used PSO to optimize the kernel function parameters and penalty factor of LSSVM, and established an LRE fault detection model. In summary, this paper chooses PSO-LSSVM and CNN-LSTM as representatives of classification and prediction algorithms, respectively, to compare their effectiveness with the proposed algorithm. This paper uses 20 sets of simulation data with different configurations obtained by randomly sampling the configuration parameters to average the results of their detection metrics, as shown in Table 2.

In order to verify the robustness of the algorithm to noise, considering the harsh measurement environment of the engine, we add 5% and 15% measurement noise to the original engine simulation model. And the engine measurement data with noise are fused to check the robustness of the algorithm to noise, and the results are shown in Table 2.

This paper focuses on the real-time and accuracy of rocket motor anomaly detection, so the selection of indicators should be in line with the theme of this paper. This paper evaluates and compares the performance of the algorithms using Accuracy, Detection Time, and mean Intersection over Union (mIoU) [36]. The three metrics are as defined in Equations (25) and (26). Accuracy reflects the ability of the algorithm to detect correctly, while Detection Time reflects the sensitivity of the algorithm to faults. mIoU serves as a reasonable and intuitive metric to clearly demonstrate the levels of false positives and false negatives.

(25) $$\begin{array}{c} Accuracy=\frac{TP+TN}{TP+FN+FP+TN}\\ mIoU=\frac{1}{2}(\frac{TP}{FN+FP+TP}+\frac{TN}{FN+FP+TN})\\ T:True,F:False\\ P:Positive(Fault)\\ N:Negative(No\;fault) \end{array}$$

(26) $$t_{A}=t_{\det\mathrm{ected}}-t_{\mathrm{fault}}$$

where $t_{\det\mathrm{ected}}$ represents the time when the fault was detected and $t_{\mathrm{fault}}$ represents the time when the fault occurred.

From Table 2, the algorithm proposed in this paper has better accuracy, which can effectively meet the accuracy requirements of the rocket power system detection. Since the proposed algorithm does not use a neural network model, this paper does not separately compare the computation time based on computational complexity. In this paper, the detection time is used to evaluate the missed detection of the algorithm, and from Table 2, the detection time of the proposed algorithm is shorter and can meet the real-time requirement of rocket fault detection. While detecting the data with noise, the algorithm’s accuracy remains at a high level, and the detection time remains almost unchanged, representing the algorithm’s robustness to the noise of the parameters.

In addition, the algorithm has false alarms and missed detections. We believe that false alarms occur because of the large fluctuations and oscillations in the rocket engine data, which are partially reflected in the fusion data, ultimately leading to false alarms.

As for the missed detection, this paper uses the detection time as an indicator, which can indirectly reflect the missed detection of the algorithm. The reason that part of the faults is not detected is that, in order to balance the false alarms and missed detection, the threshold design of the adaptive CUSUM algorithm in this paper is more conservative, which leads to the increase in the detection time.

Finally, it is worth noting that the algorithms in this paper are designed for mainstream rocket launcher models as well as configuration schemes. Different rocket architectures, dimensions, and operating conditions mainly affect the size and form of data changes, while having less impact on fault characterization. The proposed algorithm is the optimal fusion parameter obtained after training and optimization by collecting the data of the rocket, and the variation of the rocket parameters obeys its design and physical laws. Therefore, the algorithm in this paper has some generalization by collecting rocket data from different cases for training. But due to the limitation of data collection, this paper only carries out validation experiments on the rocket parameters under different faults. In subsequent research, we will continue to explore the program design of the rocket anomaly detection algorithm in various situations.

## 5. Conclusions

This paper proposes an anomaly detection method for data fusion in rocket power and control systems. The method addresses the insufficient observation information within the power system in real situations and fuses data from other systems at the data level to realize fast and accurate anomaly detection. In this paper, we design a data processing scheme based on convex optimization, which first constructs a convex quadratic programming problem, trains with preprocessed data inputs to find the optimal fusion of multi-sensor data, and utilizes an adaptive CUSUM detection algorithm to achieve fast and accurate anomaly detection. The advantage of this algorithm is that it can recognize the optimal fusion of information so that the information from multiple sensors can be most effectively utilized, ensuring rapid and accurate detection. The proposed algorithm is tested and validated on a rocket simulation platform, and the results show that the fused data can strengthen the differences in the characteristics of the data before and after the fault, improve the accuracy by 17.7% compared with the data before fusion, and reduce the detection time by 24%. In addition, the proposed algorithm has a higher accuracy and faster speed than similar anomaly detection algorithms. This paper only tested the algorithm’s performance under several similar faults. We did not consider the design of anomaly detection algorithms under all faults. We will continue to explore the performance of the algorithms under different types of faults in subsequent work.

In this paper, the anomaly detection study is mainly carried out for the internal faults of the rocket engine. The external faults of the engine may also lead to parameter changes, which requires the design of targeted algorithms to identify faults. In addition, although the proposed algorithm can be trained to improve the generalizability of the algorithm, the direct transferability of the algorithm to launch vehicles with different characteristics or configurations remains to be investigated. In summary, future work should be carried out to improve the performance and scalability of the algorithm.

## Figures and Tables

**Table 1:** The optimal fusion weight.

Para	$n_{tppo}$	$Q_{fvalve}$	$Q_{ovalve}$	$\delta_{\gamma }$
Value	0.1652	0.3044	0.0856	0.1723
Para	$\delta_{\varphi }$	$\delta_{\varphi }$	$n_{y}$	$n_{z}$
Value	0.1245	0.0647	0.0018	0.0815

**Table 2:** Comparison of algorithmic effects.

Algorithm	Accuracy	MIOU	Detection Time
PSO-LSSVM	92.9%	86.7%	1.88 s
CNN-LSTM	94.3%	89.0%	2.03 s
Single-parameter CUSUM	85.2%	74.2%	1.48 s
proposed	98.7%	97.4%	1.12 s
Proposed (with 5% noise)	98.6%	97.1%	1.15 s
Proposed (with 15% noise)	95.5%	91.3%	1.14 s

## Data Availability

Data are unavailable due to privacy or ethical restrictions.
